# Fibrotic content of LV myocardium quantified by CMR characterizes left atrial sizes and total left atrial emptying function incremental to LV functional parameters and LV myocardial mass index in patients with hypertrophic cardiomyopathy

**DOI:** 10.1186/1532-429X-14-S1-P159

**Published:** 2012-02-01

**Authors:** Yucheng Chen, Carolyn Ho, Eri Watanabe, Damien Mandry, Michael Jerosch-Herold, Raymond Kwong

**Affiliations:** 1 Cardiology Division, Brigham and Women's Hospita;, Boston, MA, USA; 2 Radiology Department, Brigham and Women's Hospital, Boston, MA, USA

## Summary

Fibrotic burden of LV myocardium quantified by CMR provides additional information about LA size and mechanics than morphological severity of LV hypertrophy measured by LV mass index alone.

## Background

Progressive myocardial stiffness of the LV in patients with hypertrophic cardiomyopathy (HCM) impedes early diastolic filling and left atrial (LA) emptying functions. Over time, LA of patients with HCM progressively dilates and undergoes structural and functional remodeling with serious cardiac events such as development of atrial fibrillation and diastolic heart failure despite optimal medical therapy.

## Methods

We studied 65 HCM patients (46 men, mean age 39±14years, mean LVEF 63±9%) with CMR. The fibrotic content of the LV myocardium (FIMeanSeg, averaged over 18 segments) was obtained using serial R1 mapping by cine-IR method before and up to 30 minutes after contrast injection. LA volumes and LA emptying function across 3 phases of the cardiac cycles, and LV function were also quantified. We sought to determine the strongest variables that predispose the patients to have LA dilatation and impaired LA emptying function.

## Results

**Figure 1:**
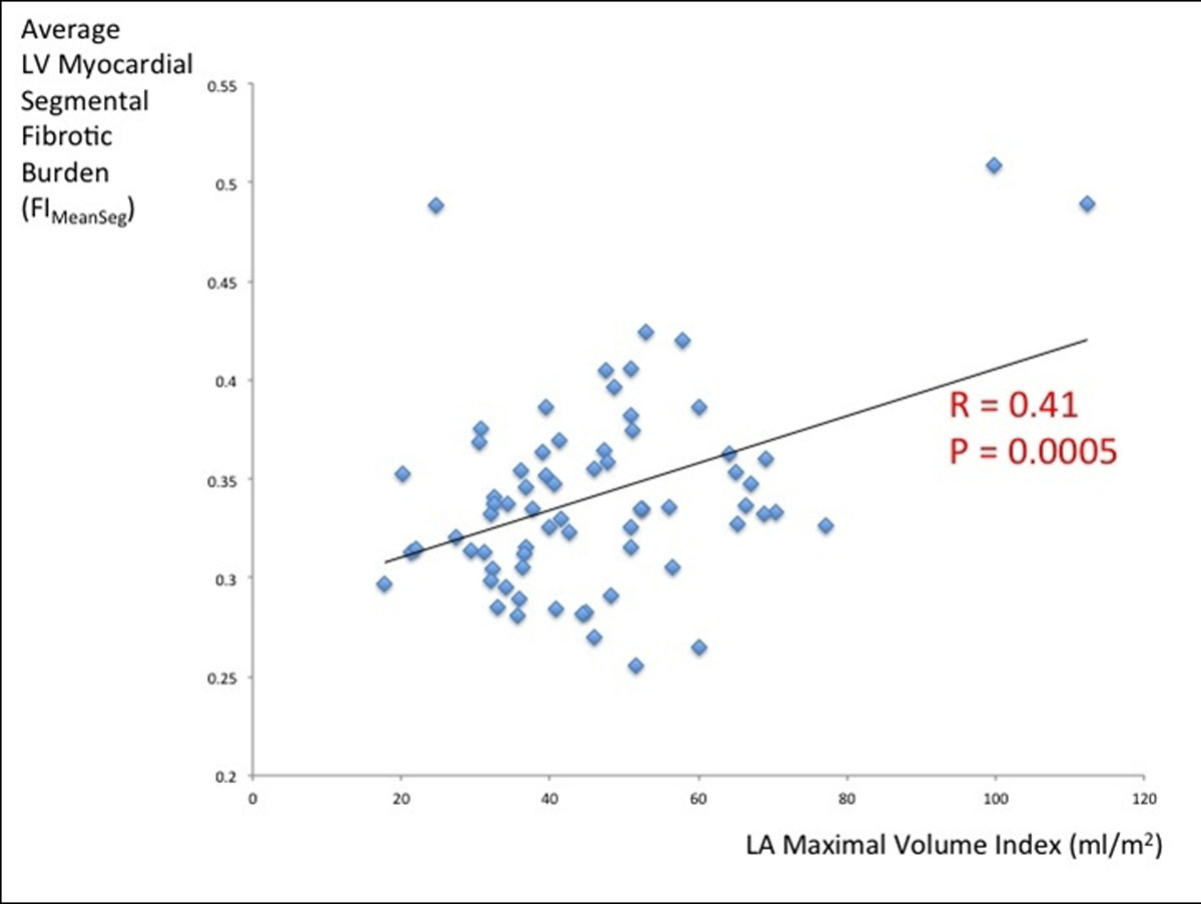


FIMeanSeg demonstrated strong positive correlation with LA volumes across all 3 phases of the cardiac cycle, with the strongest correlation with LAMax volume at end-ventricular systole (r=0.41, P = 0.0005, Figure 1). FIMeanSeg also demonstrated significant negative correlations with total and contractile LA emptying functions (r=-0.25 and -0.27, P=0.04 and 0.02, respectively). Stepwise linear regression selected FIMeanSeg with LVEDV and LVESV as the best-overall model for LA size (Table 1). FIMeanSeg was the strongest multivariable covariate in the best-overall model for total LA emptying function, with substantial incremental value over LV mass index (Table 1).

**Table 1:** Correlation between LV fibrotic index and left atrial size or function

Best Overall Model for Maximal Left Atrial Size		
	F-value	P-value

FIMeanSeg	13.22	0.0006
LVEDV (ml)	6.34	0.01
LVESV (ml)	18.89	0.0002



Best Overall Model for Total Left Atrial Emptying Function		

	F-value	P-value

FIMeanSeg	8.26	0.0055
LV mass index (g/m2)	6.50	0.01

## Conclusions

FIMeanSeg provides independent association with LV volume beyond knowledge of ventricular functional parameters and severity of ventricular hypertrophy by LV mass. This novel method may add to the current characterization of impaired diastolic function in HCM patients and may have prognostic implications.

## Funding

Yucheng Chen got the salary from China Scholarship Council.

